# Young Adults in Endemic Areas: An Untreated Group in Need of School-Based Preventive Chemotherapy for Schistosomiasis Control and Elimination

**DOI:** 10.3390/tropicalmed3030100

**Published:** 2018-09-05

**Authors:** Harrison K. Korir, Diana K. Riner, Emmy Kavere, Amos Omondi, Jasmine Landry, Nupur Kittur, Eric M. Ndombi, Bartholomew N. Ondigo, W. Evan Secor, Diana M. S. Karanja, Daniel G. Colley

**Affiliations:** 1 Centre for Global Health Research, Kenya Medical Research Institute, Kisumu 40100, Kenya; cheriyotharrison9@gmail.com (H.K.K.); awinoemmy01@gmail.com (E.K.); amosomondi2@gmail.com (A.O.); emakuto@gmail.com (E.M.N.); ondigo2002@gmail.com (B.N.O.); diana@cohesu.org (D.M.S.K.); 2 School of Public Health, Department of Biomedical Sciences, Maseno University, Kisumu 40100 Kenya; 3 Center for Tropical and Emerging Global Diseases, University of Georgia, Athens, GA 30602, USA; dianariner@yahoo.com (D.K.R); nupur.kittur@gmail.com (N.K.); 4 Institute of Tropical Medicine and Infectious Diseases, Jomo Kenyatta University of Agriculture and Technology, Nairobi 56100, Kenya; 5 Albany Medical College, Albany, NY 12208, USA; landryj1@amc.edu; 6 Department of Pathology, Kenyatta University, Nairobi 00609, Kenya; 7 Department of Biochemistry and Molecular Biology, Egerton University, Nakuru 20115, Kenya; 8 Division of Parasitic Diseases and Malaria, Centers for Disease Control and Prevention, Atlanta, GA 30333, USA; was4@cdc.gov; 9 Department of Microbiology, University of Georgia, Athens, GA 30602, USA

**Keywords:** schistosomiasis, Kato-Katz, POC-CCA, young adults, soil-transmitted helminths

## Abstract

Parasitologic surveys of young adults in college and university settings are not commonly done, even in areas known to be endemic for schistosomiasis and soil-transmitted helminths. We have done a survey of 291 students and staff at the Kisumu National Polytechnic in Kisumu, Kenya, using the stool microscopy Kato-Katz (KK) method and the urine point-of-care circulating cathodic antigen (POC-CCA) test. Based on three stools/two KK slides each, in the 208 participants for whom three consecutive stools were obtained, *Schistosoma mansoni* prevalence was 17.8%. When all 291 individuals were analyzed based on the first stool, as done by the national neglected tropical disease (NTD) program, and one urine POC-CCA assay (*n* = 276), the prevalence was 13.7% by KK and 23.2% by POC-CCA. Based on three stools, 2.5% of 208 participants had heavy *S. mansoni* infections (≥400 eggs/gram feces), with heavy *S. mansoni* infections making up 13.5% of the *S. mansoni* cases. The prevalence of the soil-transmitted helminths (STH: *Ascaris lumbricoides*, *Trichuris trichiura* and hookworm) by three stools was 1.4%, 3.1%, and 4.1%, respectively, and by the first stool was 1.4%, 2.4% and 1.4%, respectively. This prevalence and intensity of infection with *S. mansoni* in a college setting warrants mass drug administration with praziquantel. This population of young adults is ‘in school’ and is both approachable and worthy of inclusion in national schistosomiasis control and elimination programs.

## 1. Introduction

The effort to control and eventually eliminate schistosomiasis has gained considerable momentum since the adoption of the World Health Assembly (WHA) Resolution 54.19 in 2001 [1] and WHA Resolution 65.21 in 2012 [2]. These efforts have largely been by primary school-based implementation of preventive chemotherapy through annual or biennial mass drug administration (MDA) of praziquantel (PZQ) along with albendazole or mebendazole for treatment of soil-transmitted helminths (STH; *Ascaris lumbricoides*, *Trichuris trichiura*, and hookworm). While effective in bringing down the prevalence and intensity of these helminth infections [3,4], this approach alone fails to eliminate transmission of schistosomiasis and almost universally fails to treat young adults in secondary school, college or university. The need and advantage of extending MDA to students in secondary schools have been demonstrated recently [3], and we sought to determine if the same might be true in regard to young adults attending a technical college, Kisumu National Polytechnic (KNP) located in a schistosomiasis endemic area of western Kenya. Such students comprise a population that is associated with given institutions and could be incorporated into national ‘school-based’ preventive chemotherapy programs. If ignored they may represent a danger to themselves in terms of morbidity and a danger to the control and elimination programs being rolled out across sub-Saharan Africa. Our results indicate that young adults in college or university settings in endemic areas need treatment for their schistosomiasis and their STHs.

## 2. Materials and Methods

This cross-sectional study was conducted among students and staff of Kisumu National Polytechnic (KNP), which is situated within the lakeside city of Kisumu about three km east of the city center, latitude 0°6′13.7′′ (0.1038°) south and longitude 34°46′23′′ (34.7731°) east, in western Kenya. At the time of this study KNP had 3106 students and 134 teaching and non-teaching staff. This parasitologic survey was a part of a larger immunologic study [5]. Recruitment inclusion criteria were that they must be student or staff at the college, be willing to provide stool and urine specimens, undergo validated testing and counseling for HIV at the local HIV Volunteer Counseling and Testing center and sign a detailed consent form. The consideration of HIV status was critical for the immunologic portions of the study but did not impact the parasitologic data presented herein. At least one stool and one urine specimen were collected from each participant, and when feasible, three consecutive stools were collected. Specimens in plastic containers labeled with unique identification numbers were delivered to the Centre for Global Health Research of the Kenya Medical Research Institute laboratory in cooler boxes within 3–5 h of collection for parasitologic processing.

Stools were examined for *S. mansoni* and STH eggs by microscopically evaluating two slides by the Kato-Katz (KK) thick smear method [6]. The number of *S. mansoni* eggs were counted, recorded and multiplied by 24 to determine the number of eggs per gram of feces (epg). Infection intensity was classified as light (1–99 epg), medium (100–399 epg), or heavy (≥400 epg) according to World Health Organization (WHO) guidelines [7]. Urine specimens were assayed for circulating cathodic antigen (CCA) by the point-of-care circulating cathodic antigen (POC-CCA) test, as described by the manufacturer (Rapid Diagnostics, Inc., Pretoria, South Africa). STH eggs were recorded as positive or negative.

Study procedures were approved by the institutional review boards of the University of Georgia (Protocol 2012-1-10145), the Scientific Steering Committee of the Kenya Medical Research Institute (KEMRI) and the KEMRI National Ethics Review Committee of Kenya (Protocol 2303) and reviewed by the institutional review board of the Centers for Disease Control and Prevention (CDC), which deemed CDC personnel to be non-engaged. All study participants found positive for *S. mansoni* were provided PZQ treatment (40 mg/kg body weight) and all those positive for STH were provided with albendazole (400 mg).

## 3. Results

The study population consisted of 291 students or staff at KNP who volunteered and were enrolled, consented and provided at least one stool specimen. Of these, 208 provided all three requested stool specimens. All stool specimens were examined by the Kato-Katz (KK) method [6] for the presence or absence of STH eggs, and the eggs of *S. mansoni* were counted to determine infection intensity. In addition, 276 of these participants also provided single urine specimens, and these were tested for CCA by the POC-CCA test (Rapid Diagnostics Inc., Pretoria, South Africa). The demographic and parasitologic data of the participants are given in Table 1. There were no significant demographic differences by Chi square analyses between those who provided three stools and those who provided fewer. The median age of the 291 participants was 22 years, and males constituted 45.5% of this study population. Ninety-two percent were students, 4% (*n* = 12) were staff, and the status data on the other 4% were missing.

Based on examination of the first stool by the KK method (*n* = 291), the prevalence of *S. mansoni* was 13.7% (Table 1). The prevalence based on a single urine assay by the POC-CCA test (*n* = 276) was 23.2%. The prevalence of *A. lumbricoides*, *T. trichiura,* and hookworm based on a single stool was 1.4%, 2.4%, and 1.4%, respectively. For the 208 participants who provided all three stools, the prevalence of *S. mansoni* rose to 17.8% and the prevalence of *A. lumbricoides*, *T. trichiura* and hookworm was 1.4%, 3.1%, and 4.1%, respectively. There were no significant differences in terms of prevalence or prevalence of heavy infection based on gender.

**Figure 1:**
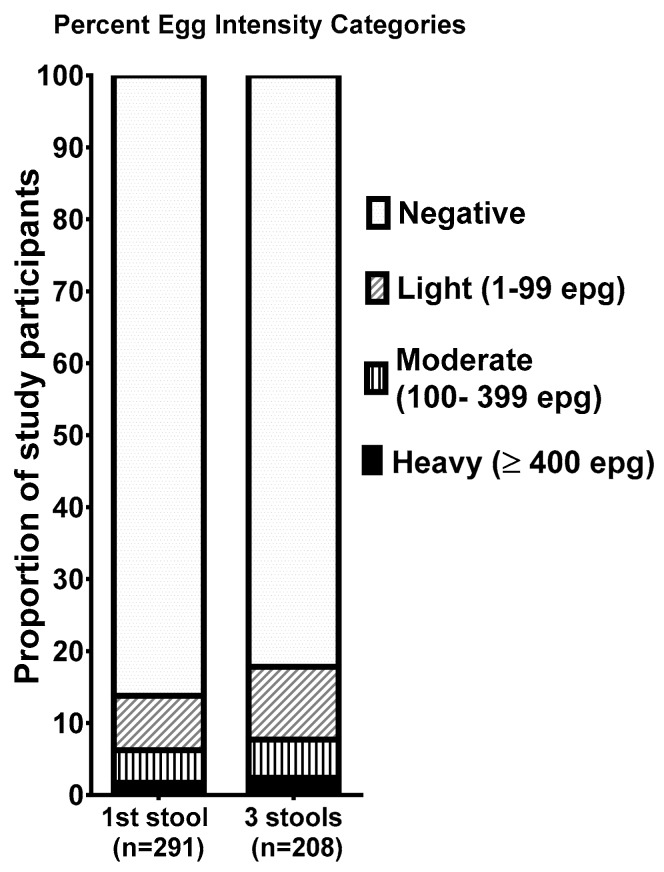
Proportion of the study participants with schistosomiasis, stratified by World Health Organization (WHO) intensity categories based on either the first stool examined (**left bar**) or three stools when available (**right bar**).

When the intensity of *S. mansoni* infection, based on the number of eggs per gram of feces (epg) was calculated based on all three stools (*n* = 208), 2.5% of this population were categorized as heavy infections (≥400 epg of stool) [as categorized by the WHO] [7] (Figure 1), 5.3% had moderate infections (100–399 epg), and 10.1% had light infections (1–99 epg). Within the *S. mansoni*-infected population 13.5% had heavy infections, another 29.7% had moderate intensity infections, and the remaining infected individuals (56.8%) had light infections. Chi square analyses showed no significant difference in *S. mansoni* prevalence or prevalence of heavy infection between students and staff (*p* = 0.765).

## 4. Discussion

We have previously shown that extending school-based MDA beyond primary schools to include secondary schools, is highly effective in rapidly dropping the prevalence and intensity of *S. mansoni* and STHs in secondary school students [3]. We have now demonstrated that there is also a need to extend national neglected tropical disease (NTD) program MDA for schistosomiasis to include college- and university-attending young adults in school-based programs in endemic areas. Such colleges and universities often have nurses staffing on-site health clinics and health science-related students who participate in health education groups, both of whom proved to be a great assistance in our study, and who could likewise be capitalized upon by national programs that wish to extend into these venues. We acknowledge that this study was conducted only at one such site and the study population was not randomly selected, but rather comprises only those who volunteered and consented to participate by providing stools and urine, as well as blood samples for the larger immunologic study [5]. Nevertheless, a substantial number of college-age students participated and a notable number of them had *S. mansoni* infections, and some of those had heavy infections.

The discrepancy between the prevalence of *S. mansoni* based on a single stool exam compared to a single urine POC-CCA assay is within the expected relative ranges of sensitivity, with the more sensitive POC-CCA always providing a higher prevalence in areas of low to moderate *S. mansoni* prevalence [8,9,10]. The increase in prevalence seen when three stools are evaluated compared to a single stool, is also to be expected, as the sensitivity increases when multiple stools from different days are tested [11,12,13].

It is clear, based on WHO guidelines, that an overall prevalence of >10% and even a prevalence of >1% heavy infection by *S. mansoni* meets the requirement for MDA in school-aged children [14]. Both of these criteria are satisfied in the population studied here although they are a different age group. While it is impossible to extrapolate and state that the same is true in other colleges and universities in endemic regions, these findings strongly suggest that this might be true and begs the question for further surveys of such populations. This is important both for the health of the infected students and in regard to control and elimination of schistosomiasis. Such heavy infections with *S. mansoni* are associated with the chronic development of severe morbidity [15], and the presence of low-to-moderate intensity infections is associated with both physical and cognitive subtle or functional morbidities [16,17]. It may be true that this population is less likely to further transmit schistosomiasis because they have more routine access to sanitation facilities; however, we previously reported that students in the KNP population who were *S. mansoni*-positive were more likely to report contact with water from Lake Victoria while in their home villages [5]. Upon returning home for holidays or other visits, they may revert to the conditions available and periodically reintroduce eggs into the environment, serving as a source of contamination that can infect vector snails and continue the life-cycle of *S. mansoni*. 

## 5. Conclusions

This study demonstrates that a substantial number of young adults attending KNP harbor *S. mansoni* and STH infections. That the prevalence of *S. mansoni* and the prevalence of heavy infections with *S. mansoni* were as high as was found in this upwardly mobile, young adult population, was somewhat surprising. It is true that KNP is in western Kenya, along the shores of Lake Victoria, an area that is known to be highly endemic for *S. mansoni,* and while its students are drawn from across the country, a majority of the student body and this study group is from western Kenya. However, the highest burden of *S. mansoni* is usually in children, and the focus of national NTD schistosomiasis programs is most often on primary school children. In an earlier study, we demonstrated that high prevalence and high prevalence of heavy infections was also to be found in secondary schools in this area and noted that, likewise, this population is usually ignored in national schistosomiasis programs [3]. While these are relatively small studies, we propose that both the secondary school and college or university populations in endemic areas need to be more extensively surveyed and included in national schistosomiasis control and elimination programs. To do otherwise is to leave a population from 14–25 years of age at risk for morbidity and as a potential risk to their communities in terms of continued transmission. In addition, in areas endemic for *S. mansoni*, surveys should consider assaying urine by POC-CCA to detect low intensity *S. mansoni* infections that may be missed by a single Kato-Katz assay. Collection of stool to test for the presence of STH as well as *S. mansoni* eggs would also be useful.

## Figures and Tables

**Table 1:** Characteristics of study participants.

Characteristic	Number of Participants (%)		
Age, median (range) years	22 (17–41)		
Male sex	132 (45.4%)		
**Prevalence of helminth infections**	**1 urine sample (CCA)** **(*n* = 276)**	**1 stool sample** **(*n* = 291)**	**3 stool samples** **(*n* = 208)**
*Schistosoma mansoni* prevalence (%)	64 (23.2%)	40 (13.7%)	37 (17.8%)
*Ascaris lumbricoides* prevalence (%)	N/A	4 (1.4%)	4 (1.4%)
*Trichuris trichiura* prevalence (%)	N/A	7 (2.4%)	9 (3.1%)
Hookworm prevalence (%)	N/A	4 (1.4%)	12 (4.1%)

Stool examinations by the Kato-Katz method; CCA assays by the point of care circulating cathodic antigen urine test.
